# Resident-Centered Outcomes in Subspecialty Neurology Education

**DOI:** 10.1212/NE9.0000000000200080

**Published:** 2023-05-26

**Authors:** Nimish Mohile

**Affiliations:** From the University of Rochester Medical Center, NY.

When I was a neurology resident, I traveled to the J. Kiffin Penry Epilepsy MiniFellowship in North Carolina, a 3-day, all-expenses-paid course focused on learning epilepsy. The aim of the course, as stated by the organizers, was to improve knowledge of epilepsy and inspire residents to pursue an epilepsy fellowship. In that short time, I improved my understanding of EEG, learned the importance of semiology, and solidified knowledge of antiseizure medications and their toxicities. I went back to residency more engaged and confident in caring for patients with epilepsy. I also made friends and learned how to play Texas hold'em. This was a respite from the daily grind of residency. To me, everything about the course was a success. Did it matter, that in the end, I did not choose to pursue a career in epilepsy and instead chose neuro-oncology?

In this issue of *Neurology*® *Education*, Charles et al.^1^ report outcomes from a similar program in movement disorders, the Focus on Common Movement Disorders (FOCMD). This 3-day course has been delivered for more than 10 years, enrolled 854 residents, and engaged more than 100 unique residency programs. These facts, alone, demonstrate consistency, reach, and longevity and are evidence of success. Pretest and posttest comparisons demonstrate an improvement in medical knowledge. Survey of long-term outcomes showed that for a majority of residents, the course influenced their decision about future career. Nevertheless, did the course achieve its intended aim of inspiring residents to become movement disorders neurologists?

Obtaining long-term follow-up data after residency is difficult for early-exposure workshops like FOCMD. Email addresses change as trainees move from one institution to the next. Residents are in some of the busiest times of their career, and simply getting time to respond can be difficult. Only 114 residents completed the FOCMD long-term survey—a subgroup that may be more engaged in the field than others. Among respondents, nearly half enrolled in a movement disorders fellowship. Although this could be seen as a demonstration of effect, how many residents would have gone down that career path anyway? It is likely, or maybe even probable, that residents who are nominated for FOCMD, accept the invitation and who chose to attend already have a penchant for the field. By so many metrics this course is a clear success, but whether it influences or changes career choice remains uncertain.

How, when, and why residents choose a particular subspecialty is a matter of consequence, particularly for outpatient subspecialties. Neurology residents increasingly see fellowship as a necessity. In a survey of adult neurology residents, 93% indicated their plans to pursue fellowship training, and options for subspecialty careers seem to be growing exponentially.^2^ The Resident and Fellow Section of *Neurology* publishes articles each year describing new emerging subspecialties such as neuropalliative care, neuroethics, or neurodevelopmental disabilities.^3-5^ Residents may not have any exposure to some subspecialties during residency training or exposure may be too late. Frequently, they must decide which specialty to choose after only 1 year of neurology-specific training. Concerned that this was placing undue burden on residents, stakeholders in education wisely sought to build consensus among neurologic subspecialties to delay and standardize the approach to fellowship applications.^6^ This work remains in progress, but even if all recommendations are adopted, most residents will have gaps in experience and exposure. Subspecialty courses such as FOCMD are aiming to fill that gap. While their intent may be to increase their own subspecialty ranks, it is important for us to also view these courses as a real service to residents.

In neuro-oncology, we have joined the “chase” for residents. With more than 50% of our fellowship spots unfilled, academic positions that are open for years and presence of a neuro-oncologist in a minority of US residency programs, engaging neurology residents early is an imperative. A 2-day course, modeled on those aforementioned, was created with the strategic intent to increase the number of applicants to neuro-oncology fellowship. Like FOCMD, most applicants likely have a preexisting interest and prior experience in their own departments. Are we actually growing the field or solidifying interest in those with established inclinations? The answer to this question may be less important than we think. We should be measuring success through the eyes of residents—and if we do that, specialty choice does not matter. The education of residents is more important than the vested interests of subspecialty societies, specialty boards, fellowship program directors, and specialists. Resident-centered outcomes prioritize education and are more likely to lead to the development of courses that effectively teach new information, expose residents to content that they may not see in their own programs, and provide a reprieve from burnout and improve their clinical abilities, no matter what they practice. Resident-centered outcomes lead us to not care about a resident's demonstrated interest in a field and may improve our ability to bring new, diverse, unexposed, and undifferentiated residents an opportunity to learn something new and fill gaps in their own residency programs. We can build these programs as a larger service to the profession rather than a service to the subspecialty.

I would not trade the experience I had to learn epilepsy in a retreat-like environment as a resident—I learned things that I still use today in my practice. FOCMD and similar courses have a critical and strategic place in resident training, and how we measure success needs to change.
